# Laser speckle decorrelation time-based platelet function testing in microfluidic system

**DOI:** 10.1038/s41598-019-52953-5

**Published:** 2019-11-11

**Authors:** Hee-Jae Jeon, Muhammad Mohsin Qureshi, Seung Yeob Lee, Jaya Dilip Badadhe, Heejoo Cho, Euiheon Chung

**Affiliations:** 1 Department of Biomedical Science and Engineering, Institute of Integrated Technology, Gwangju Institute of Science and Technology (GIST), Gwangju, 61005 Republic of Korea; 2 Department of Laboratory Medicine, Chonnam National University Hospital, Gwangju, Republic of Korea

**Keywords:** Health care, Biomedical engineering

## Abstract

Platelet aggregation and adhesion are critically involved in both normal hemostasis and thrombosis during vascular injury. Before any surgery, it is important to identify the number of platelets and their functionality to reduce the risk of bleeding; therefore, platelet function testing is a requirement. We introduce a novel evaluation method of assessing platelet function with laser speckle contrast imaging. The speckle decorrelation time (SDT) of the blood flowing through a microfluidic channel chip provides a quantitative measure of platelet aggregation. We compared SDTs of whole blood and platelet-poor blood, i.e., whole blood stripped of its buffy coat region, and found a marked reduction in decorrelation time for platelet-poor blood. The measured SDT of platelet-poor blood was 1.04 ± 0.21 ms, while that of whole blood was 2.64 ± 0.83 ms. To further characterize the sensitivity of our speckle decorrelation time-based platelet function testing (SDT-PFT), we added various agonists involved in platelet aggregation, including adenosine diphosphate (ADP), epinephrine (EPI), and arachidonic acid (AA). In this study, the results show that whole blood with ADP resulted in the largest SDT, followed by whole blood with AA, whole blood with EPI, whole blood without agonist, and platelet-poor blood with or without agonist. These findings show that SDT-PFT has the potential for rapid screening of bleeding disorders and monitoring of anti-platelet therapies with only a small volume of blood.

## Introduction

Platelets are components of blood that play essential roles in both pathological thrombosis and normal hemostasis^1,2^. Platelet aggregation is quickly involved in a sequential functional response, including several steps in platelet activation, at the site of a damaged wall. Various proteins are expressed when platelets are activated by exposure to the damaged vessel wall, mediating and binding platelets to the wall. Activated platelets from the sequential functional response (adhesion, shape changing, release reaction, and aggregation) form a hemostatic plug that blocks the site of injury to prevent blood loss^3–5^.

When the number of platelets is reduced or one of their functions is defective, the bleeding risk in patients also increases. Thus, a rapid identification of patients at high risk of bleeding, thrombosis, or hemorrhage is important prior to surgery. Because of the need to determine patients at risk of bleeding^5–7^, various platelet function tests have been developed. The Duke procedure was the first to evaluate the bleeding time via a small cut made in the earlobe using a lancet, but is now obsolete due to its poor standardization and invasiveness^8^. In the 1960s, light transmission aggregometry (LTA) was introduced to identify both acquired and congenital platelet disorders using platelet-rich plasma^9,10^. Though LTA is a historical gold standard, this method requires large volumes of blood and takes a long time to diagnose platelet disorders by evaluating responses to external aggregating agents, such as adenosine diphosphate (ADP), arachidonic acid (AA), collagen, and epinephrine (EPI)^11,12^. In the past two decades, more techniques for clinical application that are based on different operating principles have been developed to assess platelet function, including impedance platelet aggregometry, VerifyNow system, and platelet function analyzer (PFA-100)^11,13^. Even though these techniques have shown substantial improvement towards point-of-care testing (POCT), further development of simple and inexpensive methods requiring only small amounts of blood would allow for platelet function testing that is currently limited to specialized clinics and research laboratories to be performed in a more general clinical setting.

Lately, fluorescence microscopy and scanning electron microscopy are being used as platelet spreading tests^11,14,15^. However, these approaches have a limitation in clinical practice due to high variability in clinical and laboratory data, in addition to long preparation times^11^. More recently, microfluidics system has been adopted for simple and rapid testing, especially in routine laboratory tests, and is being researched toward possible future use in a clinical setting^12,16–18^. In this study, we combined laser speckle imaging with a microfluidic system to enable a simple and rapid assay for evaluating platelet dysfunctions that requires very low volumes of treated whole blood (~20 *μ*l). Using our optical and microfluidic system, we evaluated the speckle decorrelation time (SDT), which correlates platelet functions with various agonists involved in platelet activation levels, for whole blood and platelet-poor blood samples.

## Results

### Speckle decorrelation time difference between whole blood and platelet-poor blood

**Figure 1:**
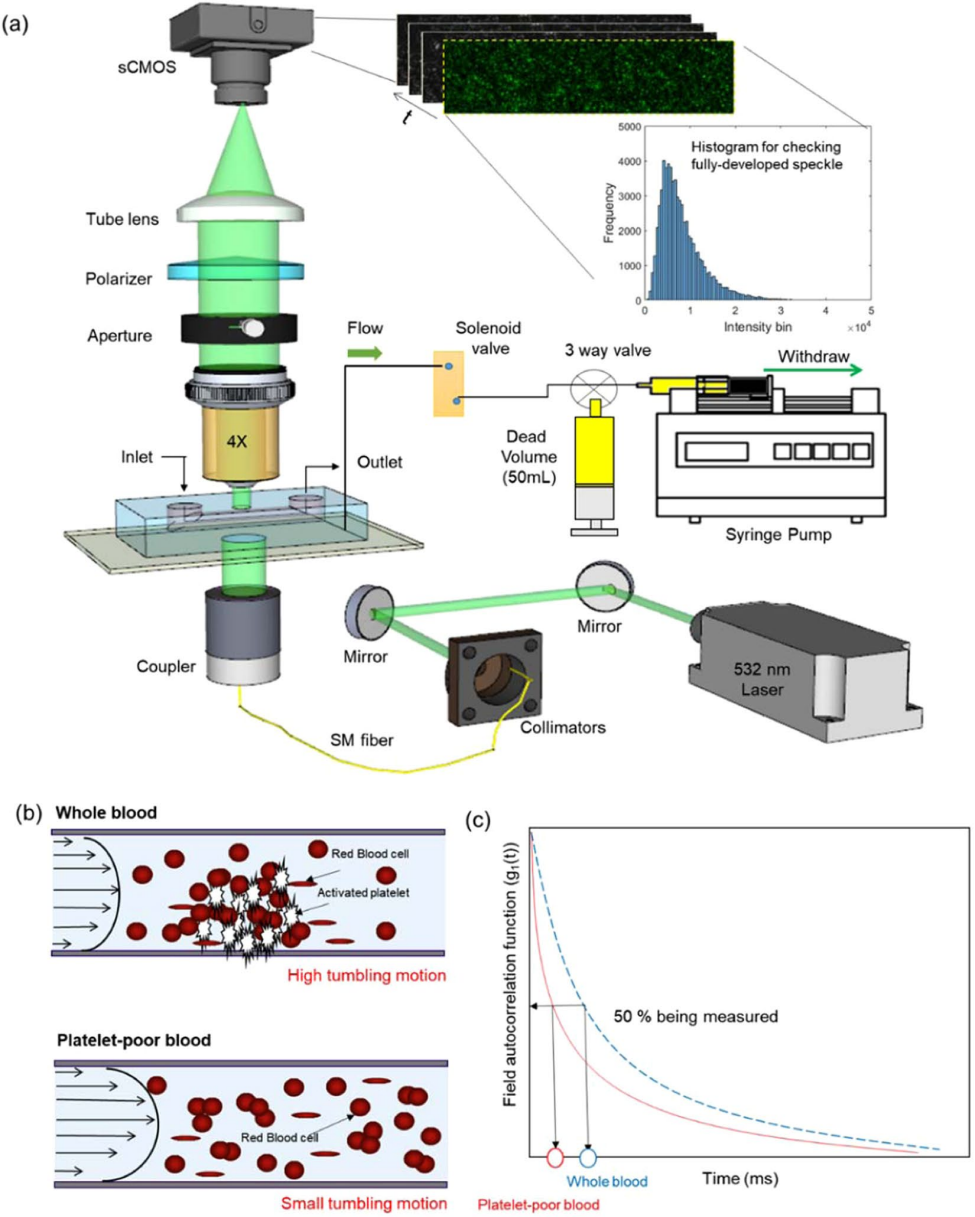
Schematic diagram to measure decorrelation times for whole blood and platelet-poor blood. (**a**) The experimental setup shows the green-color (𝜆 = 532 nm) laser light passing through the microchannel and transmitted light being collected by the objective lens of the microscope. The histogram graph shows that the mean value is 8,263 and the standard deviation is 49,818. These values indicated that our system speckles were fully developed. (**b**) Conceptual diagrams depicting the difference during flow between two types of blood samples, whole blood and platelet-poor blood, in the microfluidics channel. (Up) Fully activated platelet aggregation changes the flow condition and blood flow gets slower due to hydrodynamic resistance^18,23^. (Down) Platelet-poor blood flows faster than platelet-containing blood. (**c**) The difference in the decorrelation time between whole blood and platelet-poor blood. ND filter: neutral density filter, aperture: pinhole.

**Figure 2:**
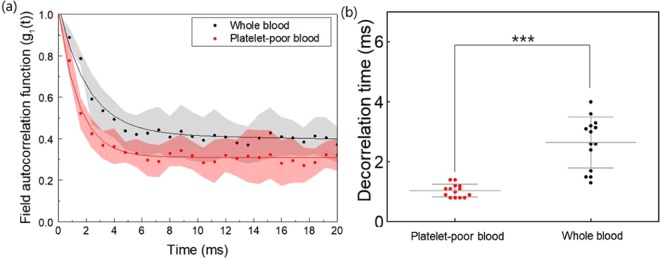
Speckle decorrelation time comparison between whole blood and platelet-poor blood. (**a**) Speckle decorrelation time comparison between whole blood (black) and platelet-poor blood (red). (**b**) Comparison of decorrelation time (n = 13 rats for each group, where each rat provided blood for both whole blood and platelet-poor blood). The field autocorrelation function g_1_(t) is calculated from the intensity-based autocorrelation function. Decorrelation time is the time for a correlation between the initial image and the subsequently captured image to drop to 50%. ***Indicates p < 0.001.

The experimental configuration for the speckle decorrelation time-based platelet function testing (SDT-PFT) is shown in Fig. 1. The speckle decorrelation curves of the whole blood (black) and the platelet-poor blood (red) are shown in Fig. 2. Because whole blood contains platelets, shear stress inside the microenvironment channel activated the platelets and blood aggregation during the pressure-gradient-induced flow. The resulting slow speed in blood flow as an effect of platelet aggregation can be observed in terms of slow changes in the speckle pattern. On the other hand, because of negligible platelet activation in platelet-poor blood, the speckles changed relatively fast. The SDTs with standard deviation for the whole blood and platelet-poor blood were 1.04 ± 0.21 ms and 2.64 ± 0.83 ms, respectively.

### Agonists-induced speckle decorrelation time

**Figure 3:**
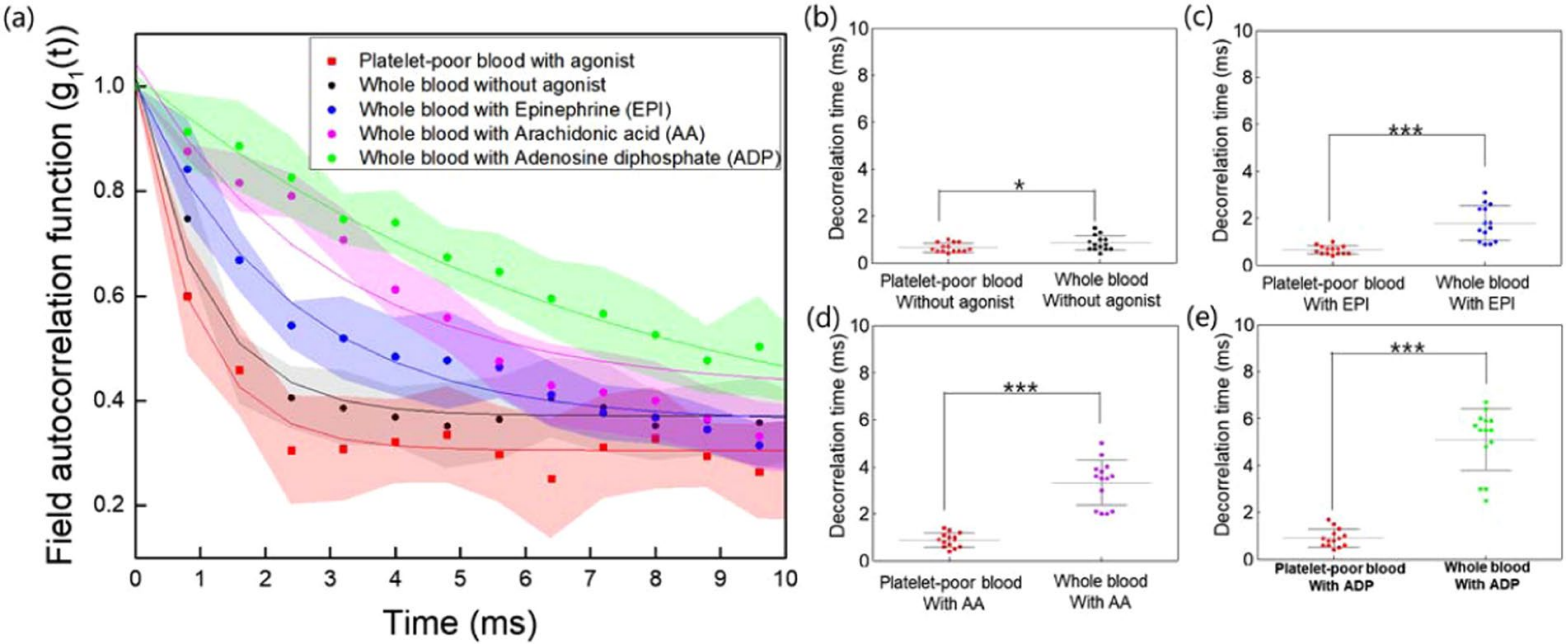
Speckle decorrelation times in response to different agonists between platelet-poor blood and whole blood. (**a**) Speckle decorrelation curves with different platelet activators (**b**–**e**) show changes effected by agonist (EPI, AA, ADP) on decorrelation times. Decorrelation times for the groups were 1.79 ± 0.71 ms, 3.33 ± 0.93 ms, and 5.1 ± 1.28 ms, respectively. Concentrations of EPI, AA, and ADP were 10 μmol/L, 0.5 μg/mL, and 10 μmol/L, respectively (n = 13). *Indicates p < 0.05, **indicates p < 0.01, ***indicates p < 0.001.

Furthermore, we also investigated the effect of drugs on platelet activation in terms of laser SDTs. The SDTs with the field autocorrelation functions are shown in Fig. 3(a). It was clearly observed that whole blood containing various agonists resulted in longer SDTs than those of whole blood without agonists and platelet-poor blood containing agonists. These agonists are potentially effective in the activation of platelets in whole blood to varying degrees^13^. However, in platelet-poor blood where the buffy coat has been removed, the agonist may have minimal effect on the blood flow in the microchannel, which was observed as a lack of change in the SDTs (Fig. 3(b–e), n = 13 rats). Platelet-poor blood with any drug has almost similar SDT value. On the other hand, the SDTs of the whole blood samples, after addition of one of the agonists EPI, AA, and ADP, were significantly increased for all tested agonists (Fig. 3(c–e)). While the SDT of whole blood without agonists, in terms of mean with standard deviation, was 0.86 ± 0.3 ms, and the SDTs for whole blood with one of the three different agonists (EPI, AA, and ADP) were 1.79 ± 0.71, 3.33 ± 0.93, and 5.1 ± 1.28 ms, respectively.

### Light transmission ratio difference

**Figure 4:**
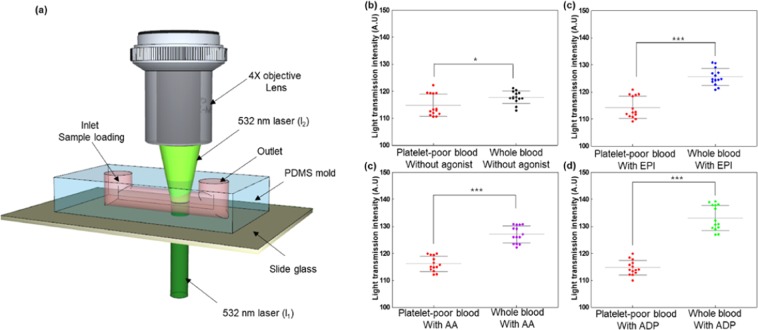
Light transmission ratio difference depending on addition of agonists to whole blood or platelet-poor blood. (**a**) The schematic for measuring transmission light intensity (l_1_ is initial light intensity and l_2_ shows the light intensity through the channel filled with each sample). Each figure shows a different condition applied on the whole blood and platelet-poor blood samples: (**b**) without agonists, (**c**) with epinephrine (EPI), (**d**) with arachidonic acid (AA), and (**e**) with adenosine diphosphate (ADP) (n = 13 rats). *, **, ***indicate p < 0.05, p < 0.01, p < 0.001, respectively.

Because platelet-poor blood tends not to form plugs even with the presence of agonists, we also measured the transmission of laser light through the microfluidic channel in which the sample flowed (Fig. 4(a)). Here, a higher light transmission ratio indicates more blood clotting^19^.

Light transmission ratio, determined by analyzing the image via the sCMOS camera, was higher for the whole blood samples, with or without agonists, compared with for the platelet-poor blood samples. In whole blood, light transmission ratio increases as the activated platelets start to form an aggregation, and will vary depending on the platelets’ aggregation size (see Supplementary S2). Addition of agonists to the whole blood samples resulted in greater platelet activation, resulting in further coagulation and increase in the overall light transmission. In the platelet-poor blood samples, on the other hand, minimum coagulation of blood resulted in less light transmission. The light transmission ratios for the whole blood with agonists, EPI, AA, and ADP were 118.0 ± 2.17, 121.5 ± 2.99, 127.1 ± 3.05, and 133.1 ± 4.5, respectively.

## Discussion

We demonstrated that the quantitative measurement of laser speckle intensity fluctuation in terms of decorrelation time could provide a means to evaluate platelet function by use of a custom-designed microfluidic channel through which the blood is passed. Using SDT-PFT, we also evaluated the effects of agonists such as arachidonic acid (AA), adenosine diphosphate (ADP), and epinephrine (EPI) for selective platelet activation. Each agonist has an effect on a different platelet activation pathway and results in different levels of platelet aggregation. Compared with the results of previous research^20^, our SDT demonstrated a similar tendency.

The SDT was calculated from the field autocorrelation function g_1_(τ) based on the sequence of laser speckle images over time. Ideally, the curve for g_1_(τ) decays from 1 at τ = 0 to zero as τ → ∞. However, during an experiment with blood in a microfluidic system, underdeveloped speckles and smeared speckles, because of experimental noise or speckle, would not allow g_1_(τ) to decay to zero^21^. In our case, we used fresh blood samples with different conditions regarding the platelets. Furthermore, an imaging region of interest (ROI) was chosen near the outlet of the microfluidic channel where the platelets got activated. Therefore, because of the aggregation of platelets, the blood cells tended to adhere to the walls of the microfluidic channel. These particles form a static optically diffusing layer, causing an asymptotic nonzero value of g_1_(τ) for all the speckle pattern time series images.

In addition, our system allows the measurement of transmitted light intensity through a method similar to light transmission aggregometry (LTA). The system is described in Fig. 4(a). LTA offers to evaluate platelet reactivity from the relative changes in the transmitted light intensity with the addition of agonists^22^. LTA measure the extent of light changes in platelet-rich plasma or whole blood, because platelet aggregation depends on factors such as the presence of agonists. Our system can measure the change in light transmission intensity similarly to the LTA system but with a very small sample volume of 20 *μ*l. Furthermore, when compared with the LTA result, there was a similar difference in light transmission intensity between whole blood and platelet-poor blood^11^.

Because the coagulation of the blood could not be directly observed in our microfluidic device, we investigated how the particle size, viscosity, and flow rate affect the SDT (see the Supplementary Fig. S1), in order to increase the reliability of this study. The removal of buffy coat, which contains large mononuclear cells and platelets, decreases platelet aggregation and blood clotting formation. We indirectly demonstrated that the decorrelation time was decreased by a reduction in the particle size. Similarly, SDT was reduced as the concentration of particles decreased. The cell count itself may be thought to be reduced by the removal of the buffy coat from the whole blood. Considering the above two cases, the decrease in the SDT, achieved by removing the buffy coat region from the whole blood, may be interpreted as an effect of cell count reduction rather than of the decrease in the unit platelet individual function itself. Further, with the addition of agonists to the whole blood sample, the SDT increased because of the resulting enhanced clot formation. We may infer that decorrelation time substantially changed because of blood clotting formation at the platelet activation level. Therefore, SDT is indirectly confirmed to be affected by a loss of platelet function. In addition, previous studies have shown that blood flow velocity varies with blood coagulation level^18,23^. Thus, changes in SDT involve these complex factors.

Even though the laser speckle approach to aggregation underscores the diagnostic potential of this technique, this method warrants further investigation in clinical areas. For example, clinical patient samples with inherited platelet dysfunction or acquired platelet dysfunction, such as from drugs including aspirin and clopidogrel, will provide more information about the potential of this approach. In this study, the platelet-poor blood was defined as whole blood from which the buffy coat was removed. Because other mononuclear cells, in addition to platelets, exist in the buffy coat region, how these mononuclear cells affect the resultant SDT is still unknown. Furthermore, the exact number of removed platelets is also unknown.

Systematically the acquisition frame rate carefully adjusted to capture the light speckle fluctuation and prevent speckle blurring with reduced contrast during low acquisition speed. In this study, rapid light speckle fluctuation was captured at a high frame rate of 1250 fps to reduce the effect of speckle blurring, because frame rate and signal to (electronic) noise ratio have a strong relationship. Even a high frame rate could potentially reduce error from speckle blurring issue^24,25^.

For the first time, we have demonstrated that laser speckle imaging with a simple microfluidics system can provide a quantitative testing of platelet function. The SDT of blood sample flowing through the microchannel was used as a measure of platelet function. We demonstrated sensitive changes in the SDTs between whole blood and platelet-poor blood, with various platelet activation conditions from the addition of agonists. Furthermore, our technique is very fast; a single test, including image capture and processing, takes less than 30 s and requires only a small volume of blood as sample. Our findings indicate that the SDT-PFT could provide a rapid and simple means for screening for bleeding disorders or monitoring antiplatelet therapies in presurgical or perioperative setting.

## Methods

### Theory for speckle decorrelation time measurement

Laser speckle is a result of interference of light after multiple scattering from an optically turbid medium, such as blood or any biological sample. This speckle pattern can vary with time as the sample changes, which was demonstrated in our experiment when the speckle pattern changed over time as the blood flowed through the microchannel. In this experiment, when blood was flowing through the microchannel, the platelets activated from the shear stress, thus changing the flow speed of the blood. With this knowledge of the conditions for platelet activation, data can be collected for time t with interval of 𝜏. The decorrelation time is calculated between two consecutive images from the time series of speckle pattern images, which were taken as the blood moved through the microchannel.

To calculate the decorrelation time, we used state-of-the-art sCMOS camera with sub-millisecond exposure time to capture the time series of 2D speckle pattern images. This arrangement is termed as multispeckle diffusing wave spectroscopy (MSDWS). In MSDWS, many speckles are measured simultaneously using an image detector, resulting in the reduction of time for measuring each individual speckle.

Because of the limitation of measuring only the intensity using silicon-based detectors such as sCMOS or CCD cameras, it is nearly impossible to measure the electric field autocorrelation function g_1_(*τ*). g_1_(*τ*) is defined as^26,27^:

1 $${g}_{1}(\tau )={\int }_{0}^{\infty }P(s)exp[(-\frac{2\tau }{{\tau }_{o}})\frac{s}{{l}^{\ast }}]$$

where *s* is the path length, *P(s)* is the path length distribution in the medium, l* is the transport mean-free path, 𝜏 is the delay time, and 𝜏_o_ is the characteristic decay time of the medium

Therefore, for the time series of speckle images, the first intensity autocorrelation function g_2_(*τ*) will be computed. Intensity-based autocorrelation function can be calculated by taking the first speckle image at a time *t*_*o*_ and then the later speckle image at time *t*_*o*_ + *τ*. The intensity autocorrelation is defined as

2 $${g}_{2}(\tau )=\frac{\langle I({t}_{o})I({t}_{o}+\tau )\rangle }{\langle I({t}_{o})\rangle \langle I({t}_{o}+\tau )\rangle },$$

where *I*(*t*_*o*_) and *I*(*t*_*o*_ + *τ*) are the intensities captured by the sCMOS at time *t*_*o*_ and *t*_*o*_ + *τ*, respectively, while the $\langle \cdots \rangle$ shows the average value of data captured for single trial on a microfluidic chip. The intensity autocorrelation function *g*_2_(*τ*) can be converted into field autocorrelation function *g*_1_(*τ*) by proper Siegert relationship^28^.

3 $${g}_{2}(\tau )=1+\beta {|{g}_{1}(\tau )|}^{2}$$

Theoretically, fully developed speckles decorrelate completely and the *g*_1_(*τ*) value goes to zero. However, in the experiment, underdeveloped and smeared speckles as well as noise have to be considered^21^.

### Development of speckle decorrelation time measurement system

Figure 1(a) shows the experimental setup. A green light laser having wavelength of 𝜆=532 nm and an output power of 50 mW (PSU-III-LCD, Changchun New Industries Optoelectronics Technology Co., Ltd., China) was used to illuminate the microfluidic chip through the multimode fiber (Thorlabs M72L02), and at the tip of the multimode fiber a coupler (Thorlabs PFA-X-2-B) is used to illuminate the sample. A microscope objective lens (Plan N 4C, NA 0.1, Olympus) focused on the surface of blood flow in the microchannel. A tube lens (focal length: 180 mm) and a linear polarizer-improving contrast by reducing the background noise in the infinity space were used in front of the sCMOS camera (Neo 5.5 sCMOS, Andor Technology Ltd. Belfast, UK). Data acquisition was done with a minimum exposure time of 0.8 ms and a frame rate of 1250 fps. For the flow of blood in the microchannel, we used a syringe pump (Pump11Elite Harvard Apparatus, US) with pulling volumes of 200 *μ*l and 400 *μ*l^18,23^.

### Platelet-poor blood preparation

In our experiment, all the animal handling followed the guidelines of the Institutional Animal Care and Use Committee (IACUC) at the Gwangju Institute of Science and Technology, Korea. All experimental protocols were approved by the GIST IACUC under protocol # GIST-2019–015. In our experiment, we implemented an interval of at least two weeks between blood collections from the same rat and used over ten male Sprague Dawley rats (12–14 weeks old with body weights between 250 and 280 g). Each time, we collected 1 ml of blood from the tail by intravenous injection of the 26G needle, from rats under isoflurane anesthesia. The blood samples from the rats were transferred to blood collection tubes (9NC 0.109M Buffered Trisodium Citrate, BD Vacutainer) for experiment^6,29^. To prepare the platelet-poor blood, the blood was centrifuged (Velocity 18R, Dynamica Scientific Ltd., UK) at 3000 rpm for 5 min, thereby removing the platelets. The buffy coat fraction of the whole blood sample was carefully removed with a micropipette^30^.

### Microfluidic system operation

**Figure 5:**
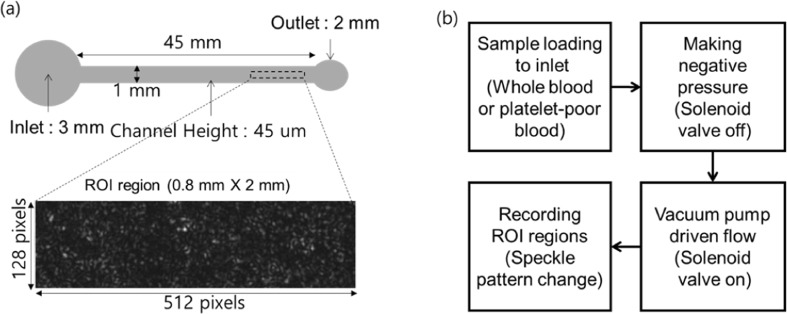
Schematic of the microfluidic channel and the procedures for collection of speckle images. (**a**) Physical dimensions of a simple microfluidics chip design and region of interest (ROI) for analysis decorrelation time changes. (**b**) Process method for operating microfluidics.

The microchip used in this experiment was custom-manufactured by soft-lithography technology as shown in Fig. 5(a). The length, width, and height of the microchannel are 45 mm, 1 mm, and 45 *μ*m, respectively. The inlet reservoir for the blood sample had a diameter of 3 mm, while the outlet for connecting to the pipette tip had an outer and inner diameter of 2 mm and 1 mm, respectively. The dimensions of the captured images were 0.8 mm × 3.3 mm (128 × 512 pixels). After collection and preparation of blood, the blood sample was poured into the inlet of the microfluidic channel. In the meantime, negative pressure was applied to the syringe pump; however, at this stage, the solenoid valve was turned off. When everything was set, the solenoid valve was then turned on, allowing the blood to flow through the channel. Every time, the center of ROI for dynamic speckle pattern imaging was set to a distance of 43 mm from the inlet in the axial direction and 0.5 mm from the side wall.

### Light transmission ratio measurement

Light transmission of waves is the movement of electromagnetic waves through a sample media. This light transmission can be affected by sample thickness and flow conditions when the light is re-reflected or absorbed by the molecules inside the sample. We also performed analysis of our data by checking the transmission light intensity and by observing the speckle pattern intensity from the camera under each sample condition. When blood samples arrived at the end of the specified ROI, sCMOS camera captured a light speckle image. To calculate the light transmission ratio of each sample, the background intensity was subtracted from the original speckle image by using a custom-written Matlab code.

## Supplementary information

Laser speckle decorrelation time-based platelet function testing in microfluidic system
