# Supplementary material for: Laser speckle decorrelation time-based platelet function testing in microfluidic system
Source: Sci Rep. 2019 Nov 11;9:16514. doi: 10.1038/s41598-019-52953-5 (PMC6848072; doi:10.1038/s41598-019-52953-5)
Supplement: Supplementary file 1 — Laser speckle decorrelation time-based platelet function testing in microfluidic system [file 41598_2019_52953_MOESM1_ESM.docx]

Laser speckle decorrelation time-based platelet function testing in microfluidic system

Hee-Jae Jeon^1, †^, Muhammad Mohsin Qureshi^1, †^, Seung Yeob Lee^2^, Jaya Dilip Badadhe^1^, Heejoo Cho^1^,
Euiheon Chung^1,*^

^1^Department of Biomedical Science and Engineering, Gwangju Institute of Science and Technology (GIST), Gwangju, 61005, Korea
^2^Department of Laboratory Medicine, Chonnam National University Hospital, Gwangju, Korea
*Correspondence and requests for materials should be addressed E. C. (ogong50@gist.ac.kr).
† These authors contributed equally to this work

**Supplementary Materials**

**Speckle decorrelation time changes with respect to particle size, flow speed, and concentration.** Supplementary Figure S1 shows the autocorrelation function curve for particle size, concentration and flow rate change. The value of the graph depends on each variation: as the bead size, flow rate, and concentration increase, the autocorrelation curve decay slowly. In this supplementary experiment, we tested the effect of scatter size and flow rate on decorrelation times as these might reflect the coagulation of the blood. We demonstrated an experiment from the microsphere particle. The electric field autocorrelation in the case of multiple scattering and Brownian motion particle diffusion can be written as

$g_{1}\left( \tau\right)=\int_{0}^{\infty} P\left( s \right)exp\left[ \left( -\frac{2\tau}{\tau_{o}} \right)\frac{s}{l^{*}} \right]$ (1) where $\tau$ is the decay time $\tau_{o}=1/(Dk_{0})$is the characteristic decay time, $k_{0}=2\pi/\lambda$ is the wavenumber of the light in the medium, D is the diffusion coefficient of the scattering particle, $l^{*}$ is the transport mean-free path, $s$ is the path length, and $P\left( s \right)$ is the distribution of path lengths in the medium. From this equation we can see that the field autocorrelation is essentially a weighted sum [weights$P\left( s \right)$] of exponential decays at rates set by $D, k_{0}, l^{*}$, and $s$. However, by examining the different flow rate for the same sample in the same experimental configuration,${P(s), k}_{0}, l^{*}$ are essentially fixed. Therefore, we can directly probe the relationship between the diffusion coefficient and the characteristic decay time. Diffusion coefficient described by the Einstein-Stokes equation:

$D=\frac{K_{B}T}{6\pi\eta r}$ (2)

$K_{B}$ is Boltzmann’s constant, T is the absolute temperature, $\eta$ is the dynamic viscosity of the medium (in our case blood) and r is the particle radius. As T are also essentially fixed during experiments, the only changes were η (viscosity) and r (radius). As expected, $g_{1}\left( \tau\right)$ decayed more rapidly for smaller particles and lower viscosity medium (Supplementary Figure S1)^1,2^.

Shear flow in turbid media is determined a correlation function that the form of the term for shear flow can be written as^1^.

$g_{1}\left( \tau\right)=\int_{0}^{\infty} P\left( s \right)\exp\left[ -2[\tau{/\tau}_{B}+{(\tau{/\tau}_{s})}^{2}]\frac{s}{l^{*}} \right]ds$ (3)

Laminar flow and Brownian motion are characterized by the shear relaxation time ${\tau_{s}}^{-1}=\Gamma k_{0}l^{*}/\sqrt{30}$ and $\tau_{B}=D{k_{0}}^{2}$, respectively, where $\Gamma$ is the mean shear rate of the scatters, $D$ is the Brownian diffusion coefficient. From this equation $P\left( s \right), k_{0},l^{*}$are essentially fixed due to the same experimental configuration. $g_{1}\left( \tau\right)$ will decays more rapidly when increase shear flow ($\Gamma)$, from above the equation.


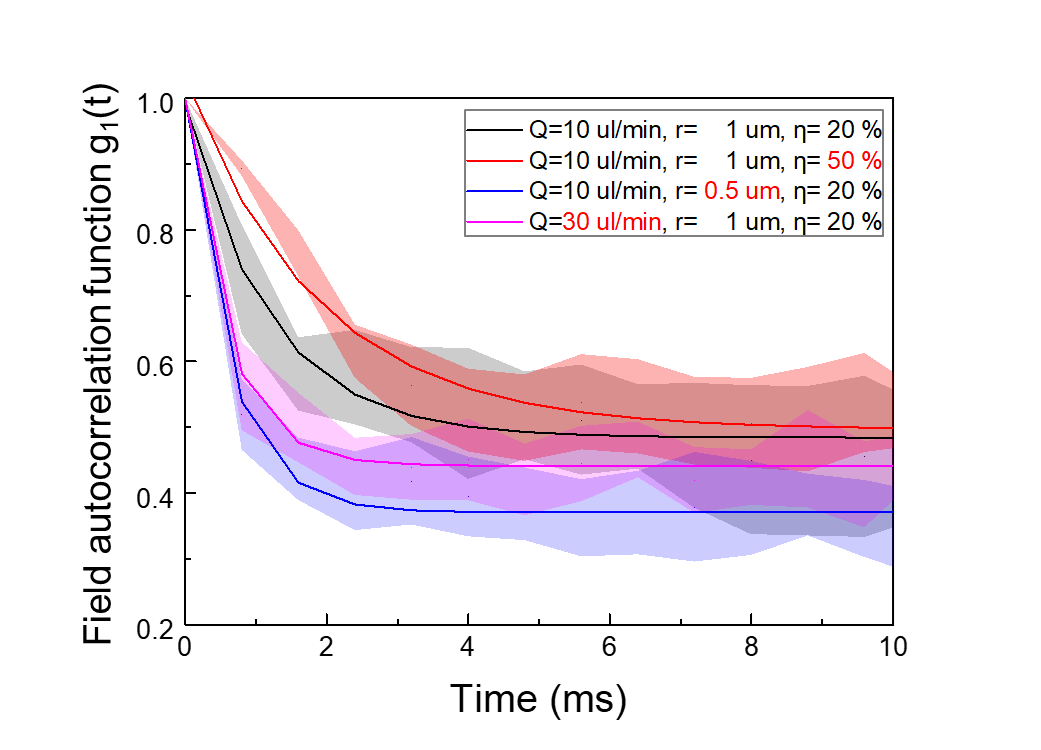


**Supplementary Figure S1. Experimental results of autocorrelation functions obtained from microsphere particle size, flow rate and concentration (n=10).** figure1 shows the autocorrelation change depending on dynamic flow rate (Q), particle size (r) and particle concentration (η). Each particles concentration was prepared from diluted DI water 2:10 (20 % =9.1 x 10^9^ particles/mL), 1:2 (50% of particle with 1 $\mu m$ = 4.55 x 10^10^ particles/mL) and 2:10 (20 % =7.28 x 10^10^ particles/mL).


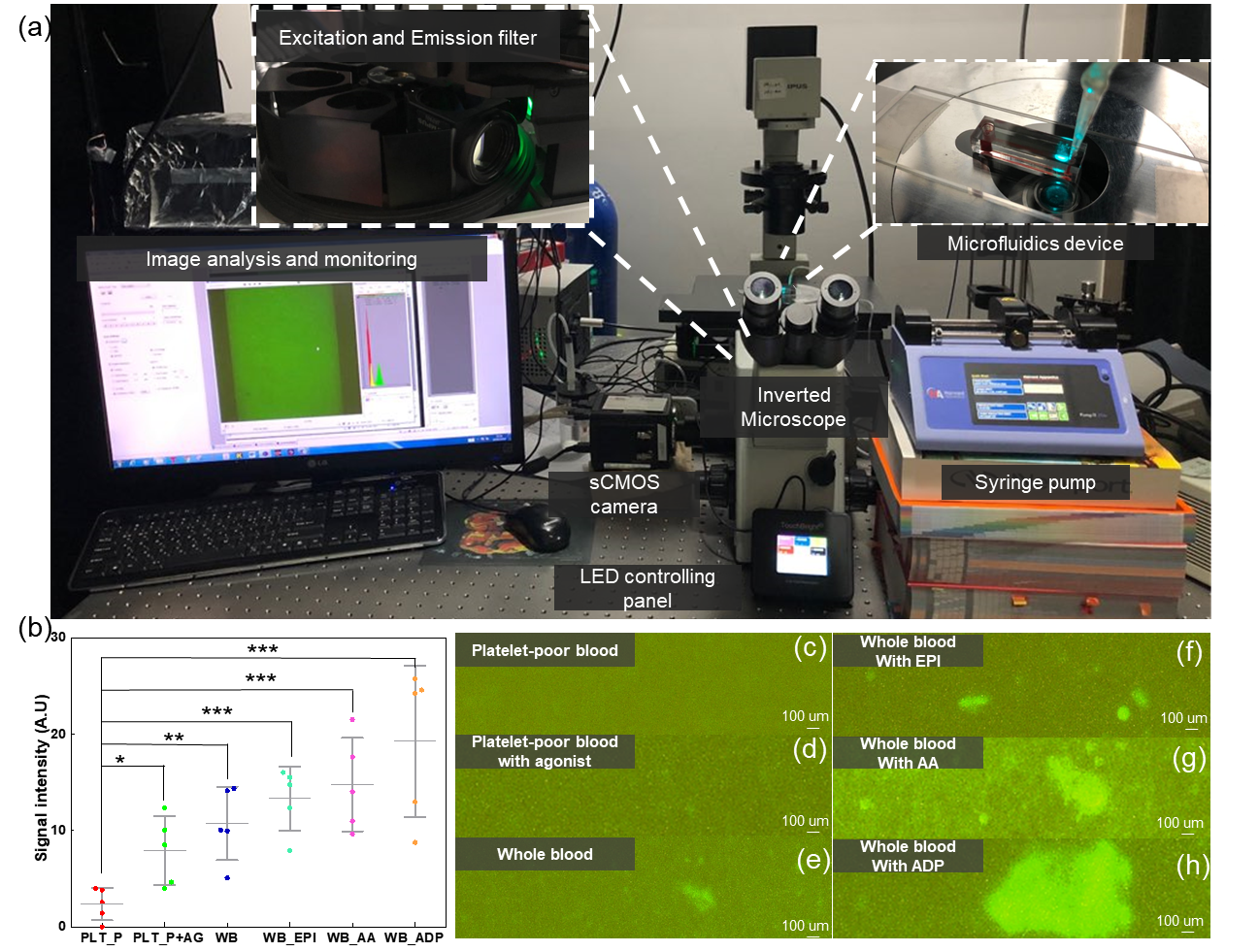


**Supplementary Figure S2. Image analysis of platelet aggregation with various conditions** Two blood samples with varying fluorescence (DIOC_6_) labeled platelet were prepared and introduced to the microfluidic device with each agonist. The sample filled in the inlet flows through the channel by negative pressure using a syringe pump. Sample-filled microfluidics channel was observed using an inverted fluorescence microscope with 20 X objective lens (UPLSAPO 20X-Olympus IX-71). The illumination regions correspond to blood aggregation by staining platelets from DIOC_6_. (a) Photography of the fluorescent microscopy measurement system. (b) measurement of the fluorescent signal intensity (c ~ h) ROI region (128 $\times$ 512 pixel) captured from a sCMOS camera (n=5). Excitation filter: 482 $\pm$35 nm Emission filter: 536$\pm$40 nm, PLT_P: platelet-poor blood without agonist, PLT_P+AG: platelet-poor blood with agonist, WB: Whole blood, WB_EPI: Whole blood with Epinephrine, WB_AA: whole blood with arachidonic acid, WB_ADP: Whole blood with Adenosine diphosphate. ), * indicates p < 0.05, ** indicates p < 0.01, *** indicates p < 0.001.

**Platelets aggregation confirmed from DIOC_6_ staining inside a microfluidic channel.** We prepared 1 mM solution of 3’-dyhexyloxacarbocyanine iodide (DiOC_6_) (#53213-82-4, Sigma Aldrich) made in distilled Deionized water. The two samples of the whole blood and platelet-poor blood each of 1 mL volume were taken in the separated citrated tube. The 2 µL solution of DiOC_6_ solution (1mM) was added to each citrated tube containing samples of whole blood and platelet-poor blood. After gently mixing the solution, the samples were allowed to incubate for 10 min at 37 °C. The two samples were introduced into the hole of the inlet part of a microfluidic device. Images were captured on a fluorescence microscope with a 20$X$objective lens. Supplementary Figure S2 (b) shows signal intensity depending on including or excluding agonists between the two samples. The fluorescence signal intensity (A.U.) for the platelet-poor blood without agonists and platelet-poor blood with agonist were 2.39 $\pm$ 1.5 and 7.39 $\pm$ 3.18, respectively. For the whole blood and blood with agonists, the fluorescence signal intensities (A.U.) of EPI, AA, and ADP were 10.74 $\pm$ 3.39, 13.33 $\pm2.97$, 14.77 $\pm4.35$ and 19.27 $\pm$ 6.99, respectively. DiOC_6_-labeled whole blood generated higher fluorescence signal intensity than DiOC6 labeled platelet-poor blood did. Signal intensity varied by adding the agonists and especially included ADP sample’s platelet aggregation are much higher than other agonists. This experiment result confirmed that there exists platelet aggregation in the whole blood and more aggregation were observed with the addition of agonists and indirectly reduce the flow rate strongly affected on decorrelation time.


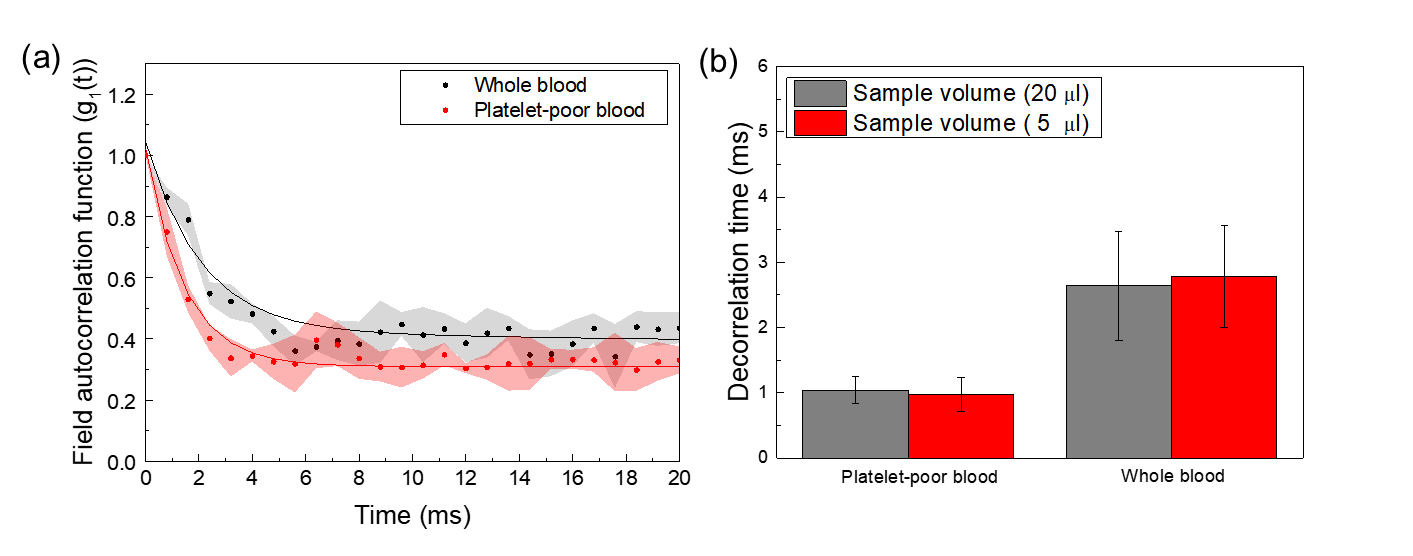
**Supplementary Figure S3. Speckle decorrelation time comparison between whole blood and platelet-poor blood with 5 μl sample volume.** (a) Speckle decorrelation curve was measured with 5 μl sample volume between whole blood (black) and platelet-poor blood (red). (b) Comparison of decorrelation time between different sample volumes with (n = 5 rats for each group, where each rat provided blood for both whole blood and platelet-poor blood). NS indicates no significant difference.

**Speckle decorrelation time measurement with reduced blood volume.** For clinical application, we attempted to further reduce the sample volume from 20 μl to 5 μl as the volume of the microfluidic channel was 2 μl. In the commercial blood glucose measuring device, a common point-of-care medical device, the capillary blood volume from a finger prick is approximately 5 μl [3]. With 5 μl volume of whole blood and platelet-poor blood, we measured the speckle decorrelation time shown in Figure S3 (a). The decorrelation time for the platelet-poor blood and whole blood were 0.98± 0.26 and 2.78 ± 0.78, respectively. Importantly, these decorrelation times were not different from those with a sample volume of 20 μl, as shown in Figure S3(b). This experimental result implies the potential for our approach with readily available and less invasive capillary blood sample. However, the decorrelation time difference of capillary blood from the finger prick method and conventional venous blood is unknown. Thus, further study with clinical samples from different blood collection will provide more information about the potential of this approach.

**References:**

1. Manikandan, S., Karthikeyan, N., Silambarasan, M., Suganthi, K. S. & Rajan, K. S. Preparation and characterization of sub-micron dispersions of sand in ethylene glycol-water mixture. *Brazilian J. Chem. Eng.* **29,** 699–712 (2012).

2. Vardhan, P. V., Suganthi, K. S., Manikandan, S. & Rajan, K. S. Nanoparticle Clustering Influences Rheology and Thermal Conductivity of Nano-Manganese Ferrite Dispersions in Ethylene Glycol and Ethylene Glycol-Water Mixture. *Nanosci. Nanotechnol. Lett.* **6,** 1095–1101 (2014).

3. Żurawska, Gajane. RESEARCH Diabetes Management. *Diabetes Manag* 6(3), 066-070 (2016).
